# Molecularly Imprinted Carriers for Diagnostics and Therapy—A Critical Appraisal

**DOI:** 10.3390/pharmaceutics15061647

**Published:** 2023-06-03

**Authors:** Emilia Balcer, Monika Sobiech, Piotr Luliński

**Affiliations:** 1 Department of Drug Chemistry, Faculty of Pharmacy, Medical University of Warsaw, Banacha 1, 02-097 Warsaw, Poland; emilia.balcer@wum.edu.pl; 2 Department of Organic and Physical Chemistry, Faculty of Pharmacy, Medical University of Warsaw, Banacha 1, 02-097 Warsaw, Poland; piotr.lulinski@wum.edu.pl

**Keywords:** molecularly imprinted polymer, diagnostic agent, targeted delivery, cancer therapy, theranostic

## Abstract

Simultaneous diagnostics and targeted therapy provide a theranostic approach, an instrument of personalized medicine—one of the most-promising trends in current medicine. Except for the appropriate drug used during the treatment, a strong focus is put on the development of effective drug carriers. Among the various materials applied in the production of drug carriers, molecularly imprinted polymers (MIPs) are one of the candidates with great potential for use in theranostics. MIP properties such as chemical and thermal stability, together with capability to integrate with other materials are important in the case of diagnostics and therapy. Moreover, the MIP specificity, which is important for targeted drug delivery and bioimaging of particular cells, is a result of the preparation process, conducted in the presence of the template molecule, which often is the same as the target compound. This review focused on the application of MIPs in theranostics. As a an introduction, the current trends in theranostics are described prior to the characterization of the concept of molecular imprinting technology. Next, a detailed discussion of the construction strategies of MIPs for diagnostics and therapy according to targeting and theranostic approaches is provided. Finally, frontiers and future prospects are presented, stating the direction for further development of this class of materials.

## 1. Introduction

Along with the growing number of emerging new methods of combating cancerous diseases, a certain information noise can be observed in the scientific literature. Numerous advancements in analytical and synthetic techniques of the past decades allowed researchers to design limitless amounts of potential therapeutic or diagnostic agents. While this increases the chances of significant discoveries, the primary focus in all such studies should always continue to improve the quality of life of patients. Regarding that, one of the most-promising fields in current medical research is personalized medicine, closely related to targeted therapy and diagnostics, which simultaneously may provide a valuable theranostic approach.

Conventional methods in cancer therapy, such as chemotherapy or radiotherapy while still used as first-choice options, very often exhibit dose-limiting toxicity. The result of such a treatment is an ineffective therapy and further deterioration of the physical and psychological well-being of a patient, which often crucially affects therapeutic success or failure. Targeted therapy promises a solution to that problem by the ability to precisely reach the neoplastic cells in the tumor microenvironment. Therapeutic agents delivered to the sites of interest possess the ability to more effectively inhibit cell proliferation and destroy cancerous tissues. Direct drug binding and the possibility of increasing the dosage of a medicine that no longer reaches normal cells reduce the adverse side effects of therapy. While very promising in theory, many studies have shown that the efficacy of targeted therapy can significantly fluctuate in different groups of patients on an individual level [1]. The explanation for that phenomenon is the heterogeneity of tumors. Diversity in their genome and phenotype causes the choice of a molecular target to be a long and complicated process, often resulting in changing targets at different stages of therapy. To overcome this obstacle, the choice of therapeutic agents should be preceded by routine genome screenings in order to obtain accurate characterizations of tumors on the microenvironmental level. That operation will lead to the appropriate selection of biomarkers further used for treatment planning and monitoring [2].

Here, one of the important aspects is therapeutic agents that exhibit a molecular recognition ability and have been identified as being of special importance in targeted therapy. Apart from the agents consisting of the drug itself, a strong focus is put on the development of drug carriers for which the introduction to the market dates back the mid-Twentieth Century [3]. The use of drug carriers allows for utilizing other types of recognizing molecules that could not be individually used as drugs due to, e.g., an insufficient toxic effect on cancer cells, as well as opens the way to the design of theranostic agents for simultaneous diagnostics and treatment or combination therapies, where the synergistic effect can improve the efficacy of the treatment.

Taking into account that fact, the idea of theranostics was implemented in medicine [4]. Over the years, the concept expanded beyond nuclear medicine, where it initially started, and became an area of interest for other fields in medical studies. The development of the field of theranostics is closely related to the many new solutions introduced in material sciences. A significant part of research concerning theranostic platforms currently focuses on nanomedical devices, mostly in regard to cancerous diseases. Nanoscale materials in medical studies offer a number of advantages that make these kinds of composites a perfect fit for reaching the tumor environment. One such quality is the ability to modify the structure or surface of nanomaterials, especially nanoparticles, in various ways and with many different compounds including targeting ligands, linkers, and drug molecules. This feature seems particularly important as it allows utilizing the active targeting approach, in additional to the enhanced permeability and retention effect (selective accumulation of nanoparticles observed due to the characteristics of tumor tissues [5]) that is demonstrated by nanomaterials and used as a passive targeting method (liposomes, polymeric micelles, dendrimers, quantum dots, and metallic, inorganic, and protein-based nanoparticles) [6,7,8]. Above that, one of the very promising branches of nanomedicine in regard to theranostic applications is radiopharmacy [9]. Current limitations that slow down the process of the clinical translation of all nanomedical platforms are mostly related to the toxicity triggered by the response of the immune system, which leads to the instability of the compound and its rapid clearance. Another containment is the manufacturing process, which should allow for the cost-effective production of materials of controlled and desired qualities, while maintaining Good Manufacturing Practice standards required by supervisory agencies and their established regulations [10].

The exploration of new drug delivery materials that offer improved transport properties, provide optimized pharmacokinetic profiles, control the drug release rate, and maintain the drug concentration within its therapeutic window, as well as enhance delivery efficacy by increasing diffusivity and biodistribution is currently one of the hot topics in medicine. Here, strong attention has been drawn to those based on molecularly imprinted polymers (MIPs) because of their advantages such as high selectivity, chemical and thermal stability, and capability to integrate with other materials, which is very important in the case of diagnostics and therapy.

In this review, the concept of molecular imprinting and its potential in therapy will be presented prior to the construction strategies of MIPs for diagnostics and therapy according to targeting and theranostic approaches. Finally, a critical outlook of synthetic feasibility and applications emphasizing the frontiers together with future prospects for that class of materials is pointed out.

## 2. Concept of Molecular Imprinting and Its Potential in Therapy

**Figure 1:**
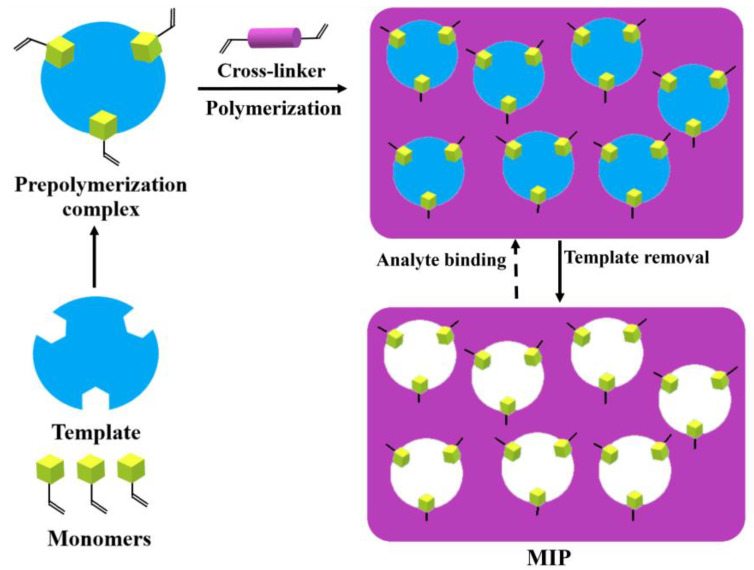
Concept of molecular imprinting.

MIPs are a class of polymeric materials prepared during a three-step synthetic process (Figure 1). In the first step, a template molecule (an imprinted compound) creates a prepolymerization complex with selected functional monomer(s), involving non-covalent interactions (non-covalent strategy) [11] or a chemical reaction is carried out to form bonds between the template and the functional monomer, resulting in a functionalized template (covalent strategy) [12]. Then, the prepolymerization complex or functionalized template is cross-linked during the polymerization process, and in the final stage, the template is removed from the polymeric matrix, either by extraction or chemical cleavage, leading to the surface modification of the resulting polymer [13].

The route to obtain a specific MIP involves the use of a template molecule that is the same compound as the target one. This means that the MIP is dedicated to the chemical compound that was imprinted, and to this compound, it should possess the highest specificity. Alternatively, the close structural analog of the compound to which the MIP shall possess specificity is often used as a template in the synthesis of MIPs. A plethora of papers describe the effectiveness of such an approach, and here, paclitaxel could be an example [14]. In the case of low stable macromolecules used as the templates, the “epitope-imprinting” process is a good alternative [15,16]. It assumes the use of a peptide as a template to form a MIP with desired specificity towards a protein. Although multiple peptides have been used as templates, the choice of peptide should be carefully screened, taking into account the antigenic domain of the corresponding therapeutic antibody that targets the receptor [17]. Moreover, the process has been adopted in the surface imprinting technique, allowing obtaining MIPs with more proper characteristics of mass transfer and sorption kinetics [18].

Up to date, MIPs are considered to be a very mature class of materials that have found widespread application in separation science and in the detection of molecules, mostly because of their properties such as high selectivity, thermal and chemical stability, and reusability [19,20]. The facile preparation together with relatively low costs have resulted in the enormous utilization of materials in the field of analytical chemistry as stationary phases in solid phase extraction and as parts of the receptor elements coupled with different detection systems [21,22,23]. It is extremely important to emphasize the advantages of MIP sensors prepared as the nanocomposites of molecularly imprinted polypyrrole film, gold nanoparticles, functionalized black phosphorus nanosheets [24], or as the molecularly imprinted polypyrrole film-coated poly(3,4-ethylenedioxythiophene)-polystyrene sulfonate-functionalized black phosphorene composite [25].

The potential of MIPs for therapy was recognized in early 2000s [26] with the idea of “intelligent drug release” and “magic bullet” drug targeting, opening significant future opportunities for the application of MIPs. Since that time, a few important papers have been published, describing the possible applications of MIPs for ocular, transdermal, or oral routes of administration. Those studies were comprehensively discussed in recent reviews [27,28,29]. It should be underlined that some ideas such as the use of molecularly imprinted soft contact lenses for ocular therapy [30,31,32], the construction of transdermal patches with MIPs for nicotine prolonged delivery [33], or studies of floating molecularly imprinted gastroretentive carriers [34] have contributed significantly to the field.

It should be mentioned that the nature of the molecularly imprinted matrix plays a crucial role in the drug release profile. Since the imprinting process itself is considered to be very complex in nature, the mechanisms of the release of drugs from MIPs could be complex, as well. Here, the swelling controlled systems such as low cross-linked hydrogels or non-swelling controlled systems such as more rigid polymer networks could be considered. In the latter systems, the relaxation of the polymer matrix governs the penetration of physiological fluid inside the net, and the transport is characterized by Fick’s laws of diffusion [35]. However, in various molecularly imprinted systems, anomalous transport mechanisms are observed because multiple complementary or sterically oriented functional residues interact with the drug, delaying its release despite the swelling degree of the polymer [36,37,38]. Finally, the mechanism that involves the release of a drug in a physical stimuli-responsive manner, for instance as a consequence of the response to a shift of physical (thermo-, solvent-, magnetic-responsive or photo-/radiation-sensitive) or chemical factors [15,34,39,40,41,42,43,44], is also considered.

Since MIPs are often described as “synthetic antibodies”, the comparison to monoclonal antibodies or aptamers should be mentioned. The biggest issue concerning MIPs in regard to the differences between these materials is still the insufficient amount of data regarding the behavior of MIPs in the biological environment. Despite that, the advantageous low cost of production, physical and chemical stability, and the wide choice of monomers for the imprinting process of MIPs altogether present a strong argument for further studies and development of MIPs for theranostics [45]. As an additional advantage compared to other drug delivery forms, such as those based on metallic nanoparticles, MIPs imprinted with epitopes, as prepared, do not require functionalization with other targeting compounds, which significantly simplifies the preparation process.

Therefore, one of the most important MIP advances is their capability for integration with various materials that could disclose additional properties such as magnetic or photochemical ones. This has opened MIPs to a broad perspective for new applications in the field of diagnostics and therapy, a field that has recently became a scientific hot topic for the exploration of MIPs [46]. Thus, the enormous progress of concepts brought the idea of “intelligent drug release” and “magic bullet” drug targeting into realization.

## 3. Construction Strategies of MIPs for Diagnostics and Therapy

The capability for the integration of MIPs with various materials has been widely adopted in the construction of MIPs for diagnostics and therapy. Here, we would like to present recent approaches in the construction of MIP-based devices for diagnostics and therapy taking into account the targeting and theranostic aspects. It has to be underlined that the presence of a MIP as an integrated part of the device (or platform) gives the opportunity to serve as the drug carriers (for instance, in cancer therapy) or to serve as ligands that recognize specific domains on the cell surface (for instance, glycoproteins overexpressed by the cancer process) [47].

### 3.1. Targeting Approaches

Since the majority of recent platforms for drug (and other compounds) delivery focus on some form of targeting strategy, two separate approaches should be highlighted, viz. active targeting, which refers to specific molecular recognition of a chosen domain that is overexpressed on cancer cells (for this purpose, different ligands binding to these domains are used), and the passive strategy, which relies mostly on the enhanced permeability and retention effect, which allows small molecules to accumulate in tumor sites. Since the terminology of passive and active targeting refers to the biological activity of a compound, here, the delivery of platforms based on their magnetic properties will be considered a different passive targeting method.

#### 3.1.1. MIPs for Active Targeting

The concept of personalized medicine emphasizes that the choice of the specific target should be based on the genetics of individual tumors, but only a finite amount of targets can be found in the recent literature. Some of the most-commonly studied cancer biomarkers such as the epidermal growth factor receptors EGFR and HER2 or folate receptor were already thoroughly described in other reviews in regard to MIPs [29,46,48]. Here, other targets recently considered in the design of MIP-based platforms are discussed.

**Figure 2:**
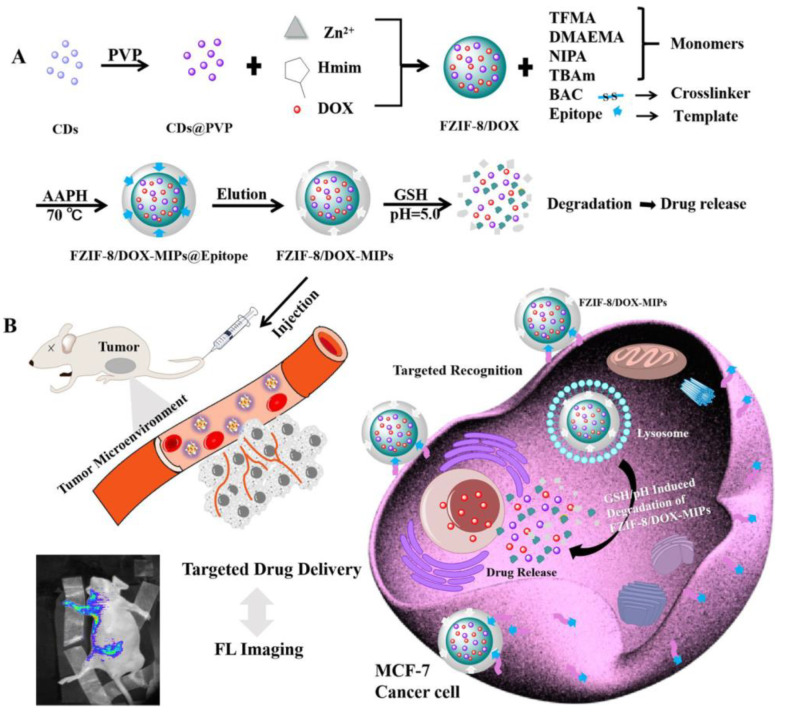
(**A**) Synthesis and gluthatione/pH dual stimulation degradation route of doxorubicin MIP-stabilized fluorescent zeolitic imidazolate framework-8 (FZIF-8/DOX-MIPs); (**B**) schematic illustration of targeted imaging and gluthatione/pH-responsive drug delivery of FZIF-8/DOX-MIPs [50]. Copyright 2020, American Chemical Society.

CD59 is a protein overexpressed in different types of tumors such as breast, lung, and ovarian cancer [49], and some recent papers on its use as a target for MIP-based composites can be found. In an excellent paper, published by Peng and co-workers [15], gadolinium-doped silicon nanocrystals were synthesized to serve as a fluorescent/magnetic resonance (MR) dual-imaging agent. Subsequently, the material was combined with (*7S*,*8S*)-3-carboxy-5-(carboxymethyl)-13-ethenyl-18-ethyl-7,8-dihydro-2,8,12,17-tetramethyl-21H,23H-porphine-7-propanoic acid (chlorin e6) prior to modification by tetraethyl orthosilicate and 3-(trimethoxysilyl)propyl methacrylate. Chlorin e6 is recognized as one of the most-commonly used photosensitizers applied in photodynamic therapy. Finally, the MIP layer was conjugated, containing two kinds of cavities after elution. The first cavity type was left by the CD59 protein’s epitope, while the other one was left to load an appropriate amount of doxorubicin, a commonly used anti-neoplastic drug for chemotherapy. In another paper, the tumor-sensitive biodegradable MIP particles with a fluorescent component based on zeolitic imidazolate framework-8 were constructed for targeted imaging and delivery of doxorubicin (Figure 2) [50]. During the construction process, fluorescent particles—carbon dots—were encapsulated together with doxorubicin in the zeolitic imidazolate framework-8 through a one-pot method. In order to stabilize the prepared core and give it targeting ability, the MIP layer with the use of the *N*-terminal epitope of the CD59 glycoprotein as the template was synthesized on the core surface. After core modification with application of trifluoromethyl acrylate (monomer), the MIP shell was synthesized from dimethylaminoethyl methacrylate, *N*-isopropylacrylamide, *N*-tert-butylacrylamide (monomers), and *N,N′*-diacryloylcystamine (cross-linker). The use of *N,N′*-diacryloylcystamine introduced into the MIP matrix disulfide bridges, which were broken under the tumor microenvironment because of the high concentration of gluthatione. Additionally, dimethylaminoethyl methacrylate, the main monomer used during synthesis, caused the protons in low pH to swell. Therefore, the MIP matrix degraded under the tumor microenvironment so that the internal part of the component containing the drug was exposed to a weak acidic environment to achieve drug release through further degradation.

Another target worth mentioning is CD47, a protein overexpressed in various cancer cells [51]. Wang and co-workers [52] used an approach combining fluorescence imaging and targeted chemodynamic therapy—a method of oncotherapy based on the use of cytotoxic hydroxyl radical converted from H_2_O_2_ by chemodynamic agents [53]. In this study, a composite consisting of fluorescent calcium peroxide as an imaging probe and a source of H_2_O_2_ and epitope imprinted polymer as a recognition unit for targeting CD47 was prepared. The MIP matrix contained copper acrylate, *N*-isopropylacrylamide, 4-vinylpyridine (monomers), and *N,N′*-diacryloylcystamine (cross-linker) residues. As the template, the exposed peptide of the extracellular domain from CD47 was used. The MIP possessing specific recognition sites enabled targeting CD47-positive cells. The cross-linker, having disulphide bonds, was reduced under the cancer cell microenvironment, then the MIP was degraded gradually, and the fluorescent calcium peroxide could contact with water in a weak acidic environment to generate H_2_O_2_. Copper ions from the MIP matrix catalyzed the production of the hydroxyl radical from H_2_O_2_ to eliminate the cancer cells. The therapeutic efficacy of the composite was tested in an in vitro model. The results showed promising biodegradable material properties, which should be further evaluated in cytotoxicity studies on cancerous and normal cells.

The carcino-embryonic antigen, a tumor marker used for diagnostic purposes in, i.e., colorectal cancer [54], was also considered in regard to MIPs for specific recognition. In a study by Han and co-workers [55], a successful synthesis of a composite based on graphene oxide, equipped with specific sites for molecular recognition of carcino-embryonic tumor markers and loaded with doxorubicin, was reported. During the preparation process, magnetic graphene oxide was obtained and functionalized with boronic acid residues. Next, the MIP, produced from dopamine used as monomer and carcino-embryonic tumor markers used as the template, was grafted onto the surface of modified magnetic graphene oxide. Lastly, the prepared material was mixed with doxorubicin to prepare the final product. The designed carrier combines several therapeutic approaches to obtain molecularly and magnetically targeted chemotherapy. The presented results also showed the pH sensitivity of the platform, which could allow for the valuable possibility of controlled drug release and, most importantly, good biocompatibility, an advantageous aspect at this stage of research. While no imaging techniques were suggested to track the obtained material, the magnetic component may be considered as a potential contrast agent for MR imaging.

**Figure 3:**
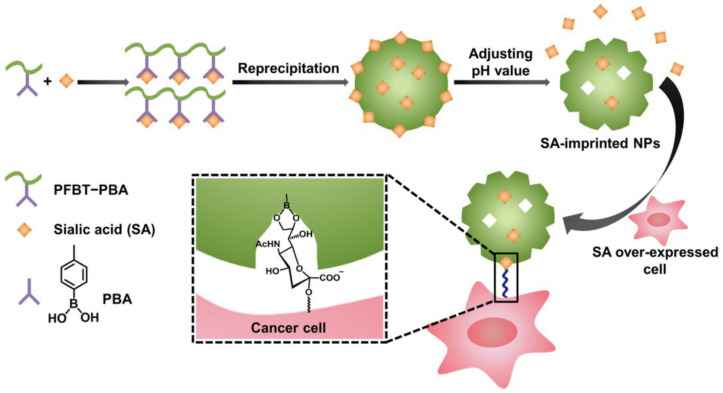
Schematic illustration of the preparation of sialic-acid-imprinted nanoparticles and mechanism of their selectivity toward cancer cells [57]. Copyright 2017, American Chemical Society.

Targeting sialic acid moieties is another approach used in the treatment of cancer [56]. As an example with regard to MIPs, the synthesis of the derivative of poly(fluorene-*alt*-benzothiadiazole) and phenylboronic acid was carried out [57]. This compound was able to provide a fluorescence response due to the presence of the fluorene and benzothiazole units, possessed a specific region to interact with the sialic acid domain by the dihydroxyborate functional group, and showed adequate hydrophilicity by the presence of quaternary ammonium salts (Figure 3). It was also tested in an in vitro model, proving a selective uptake by cells exhibiting sialic-acid-overexpressed surfaces.

#### 3.1.2. MIPs for Passive Targeting

A well-described phenomenon of enhanced permeability and retention effect shown by different types of nanomaterials is a hallmark example of a passive targeting approach. While advantageous in many cases, it comes with its own challenges, such as tackling the issue of tumor heterogeneity [58]. Its utility in regard to MIPs has been described in a different review [46], yet considering the capability of MIPs to integrate with various materials and, hence, to design more promising platforms, it does not seem to be one of the more relevant approaches.

Here, the delivery of compounds utilizing their magnetic properties could be considered another passive targeting method. In this method, once the compound containing a magnetic element enters the bloodstream, an external magnetic field is applied in order to guide the compound to the tumor site [59]. MIPs are frequently conjugated to magnetically susceptible cores such as magnetite and/or maghemite, providing a platform that responds to an external magnetic field [60]. Additionally, such platforms could be used as contrast agents in MR imaging [61]. The conjugation of MIPs is mostly carried out on the magnetite surface, modified by tetraethyl orthosilicate and 3-(trimethoxysilyl)propyl methacrylate. This step additionally protects the magnetite core against oxidation and degradation, which could be considered also a very important problem. Here, the extended studies were provided to reveal the effect of magnetic MIPs on cell survival or their degradation in a bioenvironment [62]. It was found that the degradation of maghemite MIPs in lysosome-like buffer evidenced the potential shielding effect of the polymer coating, but enzymes potentially present in the intracellular compartments modulated it. A very brilliant idea was proposed by Chen and co-workers [63], who prepared catalase-imprinted fibrous composite nanoparticles as an integrated platform for imaging and photothermal therapy. The ellipsoidal nanoplatform was composed of a magnetic β-FeOOH nanorod core and a catalase-imprinted fibrous siloxane/polydopamine composite shell. The comprehensive physicochemical analysis confirmed the structure and composition of the obtained composite. A study conducted by Parisi and co-workers [64] should also be highlighted as it proposed a new method of simplified synthesis by precipitation photo-polymerization in order to obtain magnetic imprinted nanospheres for the delivery of an anti-neoplastic drug, 9*H*-carbazole derivative. The magnetic element of the designed carrier, Fe_3_O_4_ particles, was suggested to act as a vehicle for targeted delivery through the use of an external magnetic field. While the composite showed promising results, regarding binding capacity and selectivity for the chosen drug, as well as a cytotoxic effect in cancer cells, additional biological tests would have to be conducted to confirm the compound’s safety required for potential experiments using animal models, such as stability and release studies in human plasma and viability studies on normal cell lines.

### 3.2. Theranostic Approaches

**Figure 4:**
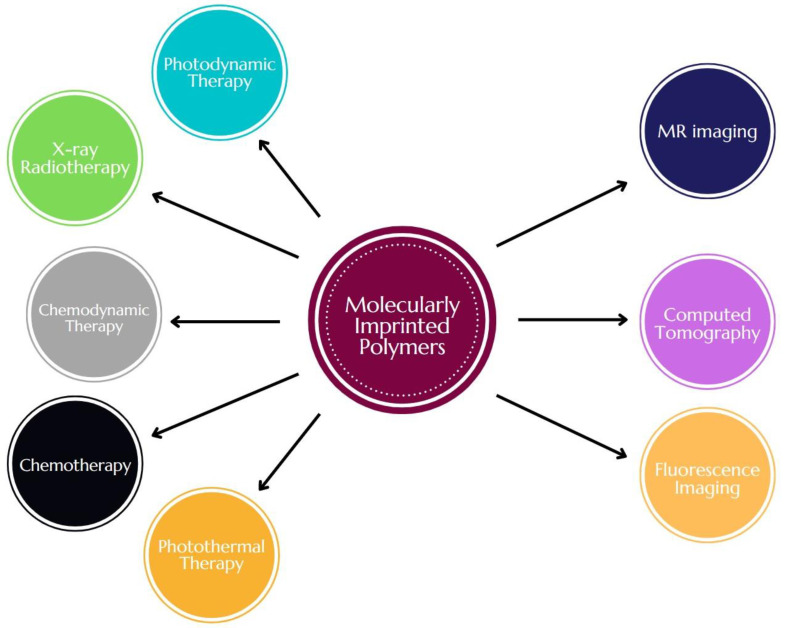
MIP application areas in theranostics.

The recent progress of research on MIPs for biomedical applications creates numerous possibilities of using MIPs as theranostic platforms aiming for various techniques (Figure 4). Current studies, concerning the above-mentioned topic to a large extent, focus on the exploitation of various MIP-based theranostic agents for fluorescent bioimaging [17,40,50,65,66,67,68,69] and were already thoroughly described in other reviews [45,46,70]. While the use of fluorescent materials in the preliminary studies on cell cultures and small animal models ensures a relatively feasible imaging method and allows investigating biodistribution in these models, the clinical application of this method for the common practice of whole-body imaging in humans is yet to be implemented. Thus, it is of great importance to explore other diagnostic methods, especially those already translated into clinical practice, regarding MIP-based theranostic platforms. Another important factor to consider is ensuring the possibility of real-time imaging during the course of treatment, which would guarantee a higher value in terms of diagnostic utility. A fitting example of such a method is MR imaging, beyond being non-invasive and offering excellent image resolution, for which numerous MIP-based composites, containing elements that may be used as contrast agents (such as iron oxide- or gadolinium-based), can be designed [71]. Additionally, MIP-based composites possessing a magnetic element can be used for precise delivery of a compound with the use of an external localized magnetic field and for utilizing the hyperthermia effect in therapy. In the case of MIP-based platforms using gold nanoparticles, another possibility is computed tomography, for which some studies have suggested utilizing gold nanoparticles as improved contrast agents [72]. Due to the significant functionalization potential of MIPs, there is also the possibility of designing composites imprinted with ligands and proteins that are feasible for radiolabeling. The presence of a radiolabeled ligand in a MIP matrix introduces the possibility of using radiotracer-based diagnostic tools such as positron emission tomography or single-photon emission tomography. Despite the potentially easy synthesis, an important issue in this case could be polymeric degradation caused by radiation emitted by the tracer; hence, radiation stability studies would have to be the first step in the evaluation of such a material. Regarding therapy, numerous reports can be found on using MIPs in delivery for chemotherapy drugs, but also as vehicles for photothermal and photodynamic therapy [29,46,73]. Here, some of the recent studies on MIP-based theranostic agents showing the possibilities of combining different therapeutic and diagnostic methods will be highlighted with a summary of chosen approaches of MIP-based theranostic platforms presented in Table 1.

**Figure 5:**
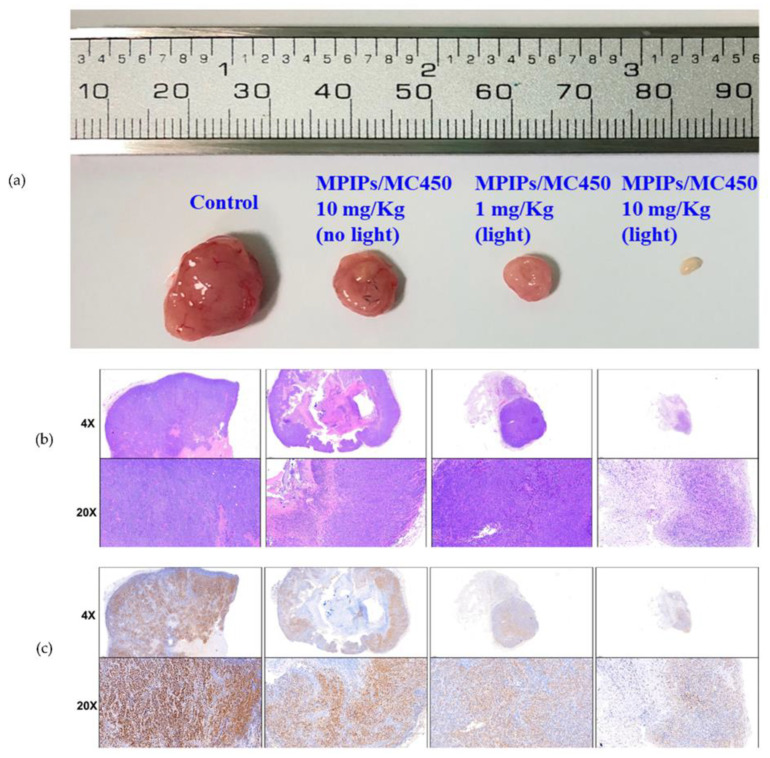
Pictures of tumor specimens from the xenograft models with the treatment of peptide-imprinted nanocomposite (**a**). Hematoxylin and eosin staining of the above tumor specimens (**b**). Immunohistochemical staining with anti-proliferating cell nuclear antigen primary antibodies (**c**) [74] (CC-BY 4.0).

In the study conducted by Lee and co-workers, magnetic nanoparticles imprinted with the peptide sequence of programmed death-ligand 1 protein were synthesized with the additional element of immobilized photosensitizer, thus introducing a novel combination of targeted photodynamic and immunotherapy [74]. A significant advantage of this study, regarding all research on the use of MIPs for biomedical applications, was including an experiment on an animal tumor model, which proved that the highest treatment efficiency was observed in the case of combined therapy (Figure 5).

**Figure 6:**
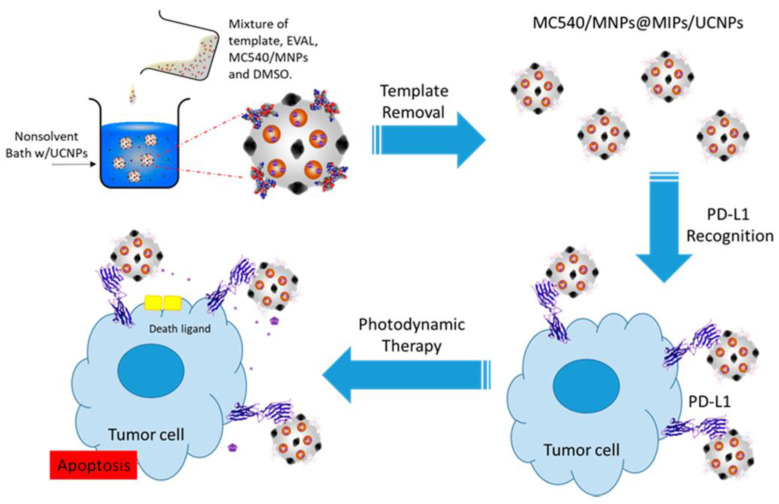
Preparation and administration of programmed death-ligand 1 peptide-imprinted composite nanoparticles [76] (CC-BY 4.0).

**Figure 7:**
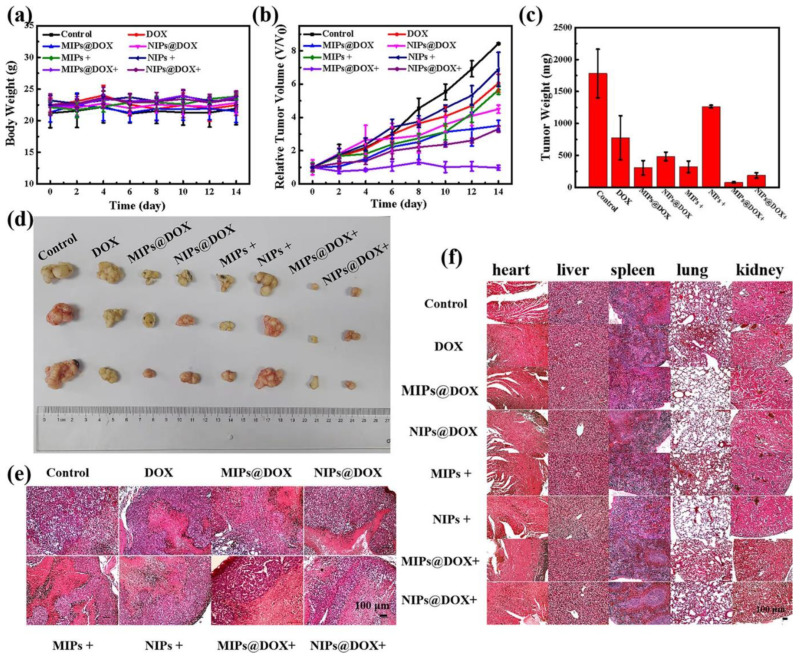
The effect of imprinted and non-imprinted drug carrier doxorubicin treatment of mice according to body weight changes (**a**), relative tumor volume (**b**), average tumor weight (**c**), and corresponding tumor tissues (**d**). Hematoxylin and eosin staining of tumor sections (**e**) and main organs (**f**). Reprinted with permission from [15]. Copyright 2020, American Chemical Society.

Exploiting the magnetic properties of the composite could be another step in the research, possibly allowing for imaging of the synthesized material in an animal model. In a study conducted by the same scientific group [76], an imprinted magnetic platform of structure and application similar to that previously described was prepared, with additional embedded upconversion nanoparticles that permit using near-infrared light in order to activate the photosensitizer included in the composite (Figure 6). A disadvantage of the composite was the demonstrated elevated cytotoxicity in human hepatoblastoma cells (HepG2) in the absence of a near-infrared light source; although, as was pointed out, this effect could be reduced by modifying the nanoparticles’ surface—a relatively easy method in the design of MIP-based materials. A different approach to combining MR imaging, fluorescence imaging, chemotherapy, and photodynamic therapy with molecular targeting was described in a study by Peng and co-workers [15]. The obtained composite was tested in vitro and in vivo on a tumor-bearing mice model, and the synergistic effect of chemodynamic and photodynamic therapy was investigated, showing the highest therapeutic efficacy in the case of combined treatment (Figure 7). In order to confirm biocompatibility of the designed platform, a histopathological analysis was conducted, proving its safety for potential in vivo applications.

Although it was suggested that the composite could be used for MR imaging, no such experiments on animal models have been described, and it would be of benefit to consider such studies in the future. In one of the most-recent papers [75], an interesting approach was described by combining MIP hydrogel nanoparticles and gold nanoparticles. The MIP-based hydrogel possesses improved biocompatibility and the potential ability to cross natural barriers, as well as to express prolonged circulation in the blood [77]. At the same time, gold nanoparticles demonstrate potential in various diagnostic and therapeutic applications [78]. Due to the presence of the specific sites for the recognition of human serum albumin, a protein corona is formed around the composite upon exposure to human serum, which ensures a stealth effect and, thus, enhanced stability in vivo. This phenomenon leads to a lower accumulation of the nanomaterial in the spleen and liver, which is a common concern in nanomedicine platforms exploiting gold nanoparticles, and was proven in the study on the mouse model of pancreatic cancer. Here, the accumulation of Au in the organs was investigated with the use of inductively coupled plasma mass spectrometry; however, the properties of gold could allow for its biodistribution measurements through computed tomography, which may provide a potential research topic in future studies. The authors of the described report further highlighted the radiosensitizing properties of gold, which increased the therapeutic efficacy of X-ray radiotherapy, where the mechanism of passive targeting through an enhanced permeability and retention effect was utilized. The key advancement regarding this study is the possibility of creating stealth nanoplatforms for targeted therapy that do not trigger a response from the immune system, and this was also recently described regarding nanogels imprinted with the fragment crystallizable domain of immunoglobulin G [79].

To sum up, the main advantages of MIPs include the possibility of producing them from various compounds and the capability of integrating them with different materials in order to create platforms with huge potential in diagnostics and therapy. More interestingly, due to the synthetic process, MIPs contain regions on their surface that are specific towards targeted domains, for instance on the cell surface. Thus, as prepared, MIPs do not have to be further functionalized with targeting ligands. This is a great advantage of MIPs that allows omitting additional steps of preparation, notably considering those materials for theranostics.

## 4. Frontiers and Future Prospects

Taking into account the frontiers in the field of MIPs for theranostics, they could be related to the effectiveness of the imprinting process, the biosafety of materials (mostly for those at the nanosize scale), and the regulatory measures that allow them to be implemented in clinical practice.

It should be mentioned that, while the imprinting process of low-molecular weight compounds looks straightforward, the use of this technology for effective imprinting of high-molecular weight compounds and macromolecules is more complicated. The formation of prepolymerization complexes with small organic molecules was proven in many studies by spectroscopic methods, such as proton nuclear magnetic resonance [80,81], and by theoretical analysis, which is a powerful tool used to support the stabilization of adducts, as well as to explain the nature of possible interactions [82]. It could be supposed that the prepolymerization complexes affect the extension of the surface of the resulting polymers, at least when highly cross-linked materials are synthesized, which was also proven by the porosity measurements [83,84]. Nevertheless, a few problems related to the conformational or isomeric stability of templates should be kept in mind while designing MIPs. Moreover, the harsh polymerization conditions, frequently used for the preparation of MIPs, can also affect the structural stability of the template [85]. Here, a careful choice of templates together with the application of more controllable polymerization techniques could be considered a solution. In our opinion, special attention should be paid to the previously mentioned epitope imprinting technique, seeming to be one of the most-promising approaches described thus far to effectively provide MIP systems specific towards macromolecules.

The polymerizable components of the prepolymerization mixture play a crucial role in the formation of the MIP. Here, various functional monomers and cross-linkers are applied to serve as compounds, forming a polymer backbone of the MIP. These aspects were closely discussed in many reviews dedicated to MIPs and will be omitted here [19,46]. The application of vinyl-derived compounds, such as methacrylic acid, *N*-isopropylacrylamide, *N,N*-methylenebisacrylamide, and ethylene glycol dimethacrylate, was verified by many scientific groups, but, in our opinion, extended investigations of naturally derived compounds are still required. From our perspective, it is important to pay special attention not only to the biocompatibility of MIPs, but also (if possible) to the biodegradability of MIPs as materials for theranostics. For example, as components, carboxymethyl cellulose–chitosan/alginate composite [86], Konjac glucomannan extracted from Konjac tubers [87], tannic acid [88], fructose [89], and glucose [90] have been examined as the potential materials to form MIPs as drug carriers. Those naturally derived compounds can easily undergo reactions to provide specific functional groups, which are limited due to their low selectivity. The biodegradability of MIPs could be provided by the application of naturally derived components, but the resulting MIPs are characterized, in general, by low selectivity. The alternative could be an application of metal–organic frameworks to provide biodegradability. In a very interesting paper, biodegradable magnesium-ion-doped silica-based molecularly imprinted nanoparticles for targeting tumor cells and drugs’ controlled release were described [91]. The construction involved a new degradable functional monomer prepared from glycerol and lactide, on magnesium-ion-doped stellated mesoporous silica nanoparticles, for the delivery of doxorubicin. The molecularly imprinted layer avoided premature drug leakage, which is considered to be an important problem of MIPs as drug carriers. Meanwhile, the large number of ester bonds contained in the functional monomers in the layer was degraded by protonation in the tumor microenvironment to expose the drugs, because of the Mg-O bond property to pH-sensitive breakage in the slightly acidic conditions [50]. Similarly, it is worth mentioning that zinc-based metal−organic frameworks can undergo complete biodegradation in an acidic environment. A different way to introduce biodegradability to the MIP is to use the well-known derivatives of poly(ε-caprolactone) [92] or poly(lactide-co-glycolide) [93]. However, this approach has been little investigated until now. Finally, the problem of the insufficient binding capacity of the MIP drug carrier should be discussed. Here, the use of a metal–organic framework was analyzed for targeted delivery of doxorubicin [94]. Porous zirconium-based metal–organic frameworks are characterized by a high specific surface area and extended porosity. Thus, conjugated with MIPs, they could provide satisfactory drug loading efficiency. Similarly, the use of polyhedral oligomeric silsesquioxanes, the organic–inorganic hybrids of a cage-like architecture, together with liquid crystal monomers was verified as efficient to provide an improvement of the physical and mechanical properties of the composite. The favorable material properties were a result of the reinforcement of the rigidity of the polymers at the molecular level [95].

Another aspect regarding the biosafety of MIPs is the very limited data related to the behavior of the material in the biological environment, a significant disadvantage compared to other competing drug delivery systems. Even though the design of novel composites of different structures brings valuable information and broadens the knowledge of the synthetic process of MIPs, it would be beneficial to focus on a thorough examination of already described materials regarding their biocompatibility and biodegradability on in vitro and in vivo models. Results from more extensive biological studies could potentially pinpoint the specific problems shared by all MIP-based platforms, which are not noticeable at the physicochemical characterization stage of research, providing the information about critical aspects of designing MIPs for medical applications.

The main challenge, in regard to designing novel theranostic agents based on MIPs but also other materials (such as nanoparticles, liposomes, proteins), remains the costly in vivo studies on animal models. Similar to any new pharmaceutical being introduced to the market, they need to be in compliance with appropriate drug administration agency standards and regulations, which means being subjected to all stages of pre-clinical and clinical trials [96]. Aside from the length and high cost of such studies, reproducibility and the ability to scale-up the whole manufacturing process are other important challenges that need to be overcome. It is preferable that all the possible issues related to production are described at the earlier stages of the new platform’s design, to ensure that not only medical, but also the economic benefits would be significant. In the case of nanovehicles, many characteristics concerning clearance and, hence, biosafety can be considered at the initial design stage through a careful choice of the nanomaterial’s size, shape, and its surface modification (leading to, for example, stealth nanoplatforms) [97]. For MIPs specifically, theoretical studies will be especially useful in this early phase. The complexity of all of the novel nanosystems seems to be currently on the rise. While the development process becomes often more complicated, it is also a trigger for new discoveries in the material sciences, and most importantly, it significantly increases the effectiveness of these materials. In regard to regulations, a big problem remains also an inefficient classification system for these new theranostic platforms, which, if revised and improved, could help in creating universal protocols, leading to better standardization of the studies involved in the new drug registration process for specific types of materials [98]. It is crucial to emphasize that, while the interdisciplinary cooperation of scientists is essential for the progress in MIP-based theranostic agents’ design, the insights from industry specialists experienced in the commercialization of drugs may be the missing aspect that will push the field forward.

The safety measures and clinical studies of new carriers are often omitted together with the long-term compatibility and clinical performance of those materials. It should become a standard in future studies, allowing those materials to be marketed in the foreseeable future. It should be emphasized that the fabrication aspects such as the reproducibility and scale-up possibilities ought to be clarified. Thus, the regulatory affairs, as well as economic aspects could be identified here as a significant barrier to commercialization.

Despite that, a wide selection of carriers with different properties intended for several imaging techniques [99] and various imprinting techniques allowing for targeted drug delivery of high specificity [100] provide a substantial potential of MIP-based platforms in biomedical applications. In the end, it should be emphasized that the statement from the previously referred to review by Sellergren and Allender, “The future will tell how far the imprinting engineers can take MIPs, and whether the fully synthetic antibody is destined to become fact or fiction” [26], is currently being realized. We are the witnesses of a process that looks very promising for the future progress of MIPs in diagnostics and therapy.

## Figures and Tables

**Table 1:** Chosen examples of different theranostic platforms utilizing MIPs.

Type of Formulation	Therapeutic Method	Diagnostic Method	Targeting Approach	Ref.
zeolitic nanoparticles as drug carriers	chemotherapy (doxorubicin)	fluorescence imaging	active targeting to CD59	[50]
Fe_3_O_4_/Fe nanorods	photothermal and radical therapy	MR imaging	magnetic-guided	[63]
core-shell nanoparticles as drug carriers	chemo- (doxorubicin) and photodynamic therapy	MR and fluorescence imaging	active targeting to CD59	[15]
gold nanorods	photothermal therapy	fluorescence imaging	active targeting to sialic acid	[67]
polymeric nanoparticles as drug carriers	chemotherapy (vinblastine)	fluorescence imaging	active targeting to folate	[69]
magnetic MIPs as drug carriers	chemotherapy (9*H*-carbazole derivative)	MR imaging (not considered in the paper)	magnetic-guided	[64]
graphene oxide-based composite	chemotherapy (doxorubicin)	MR imaging (not considered in the paper)	magnetic-guided and active targeting to carcinoembryonic antigen	[55]
magnetic nanoparticles	photodynamic and immunotherapy	MR imaging (not considered in the paper)	active targeting to programmed death-ligand 1 protein	[74]
silica nanoparticles-based composite	chemodynamic therapy	fluorescence imaging	active targeting to CD47	[52]
Au-embedded nanogels	X-ray radiotherapy	computed tomography (not considered in the paper)	passive targeting through enhanced permeability and retention effect	[75]
